# Atomic-resolution view of complete TCR-CD3 revealed

**DOI:** 10.1007/s13238-019-00677-7

**Published:** 2020-01-06

**Authors:** Jijie Chai

**Affiliations:** Institute of Biochemistry, University of Cologne, 50829 Cologne, Germany

Antigen-specific T-cell responses are triggered via interaction of T cell receptors (TCR) with pathogen- or tumor-derived peptides presented on major histocompatibility complexes (pMHC) by antigen-presenting cells (APCs). The mechanisms of TCR signaling are fundamentally important to our understanding of adaptive immunity and TCR-based therapies. This can be exemplified by the fact that studies of TCR have led to clinical development of chimeric antigen receptor T (CAR-T) cells (Srivastava and Riddell, 2015). TCR is a multiprotein complex consisting of variable TCR receptor α and β chains (TCR α/β) associated with the dimeric signaling modules CD3γ/ε, δ/ε, and ζ/ζ. Over the past two decades, many models have been proposed on the arrangement of the receptor subunits, stoichiometry of the TCR-CD3 complex and the mechanisms of TCR triggering (Rudolph et al., 2006). Our knowledge of TCR signaling, however, is far from being complete partly due to the lack of structural information of a complete TCR-CD3 complex. In one recent remarkable study published in *Nature* (Dong et al. 2019), an atomic-resolution view of a full TCR-CD3 complex has been revealed. The structure significantly advanced our understanding of the mechanism of TCR-CD3 assembly and offered unprecedented insight into TCR triggering.

The authors reconstituted a human TCR-CD3 complex using an elegant screening system. Combination of glutaraldehyde-based cross-liking and cryo-electron microscopy (cryo-EM) allowed them to obtain a structure of the complex at 3.7 Å, revealing for the first time the molecular architecture of an intact TCR-CD3 complex at an atomic-resolution. The structure showed a 1:1:1:1 stoichiometry and relative subunit positioning of TCR-CD3. The TCR α/β constant domains (TCR Cα/Cβ) and the extracellular domains (ECDs) of CD3γ/ε and CD3δ/ε’ form a trimer-like structure adjacent to the plasma membrane (PM), whereas the TCR α/β variable domains (TCR Vα/Vβ) are positioned distal to the PM (Fig. 1A). Despite the contacts made in the ECDs, assembly of the TCR-CD3 complex is mainly mediated by its transmembrane (TM) domains and connecting peptide (CP) regions between ECDs and TMs. The two TM helices of TCR α/β are surrounded by the six TM helices of the CD3 subunits via extensive hydrophobic and ionic interactions, forming an α-helical barrel-like structure that has a major role in assembling the TCR-CD3 complex. Formation of the barrel-like structure agrees with the data showing a compact assembly of the TM domains of TCR-CD3 (Krshnan et al., 2016). Interactions involving the CP regions further fortify assembly of the complex. By contrast, the intracellular tails of the CD3 subunits are unstructured, consistent with previous NMR study (Xu et al., 2008).

**Figure 1:**
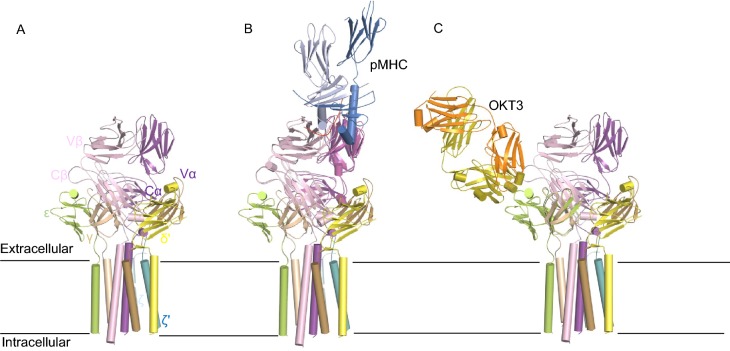
**cryo-EM structure of the TCR-CD3 complex at 3.7 Å**. (A) Overall structure of the TCR-CD3 complex. (B) Structure comparison between TCR-CD3 and OKT3-bound CDεγ. The extracellular domains of CDεγ from the TCR-CD3 complex were used as the template for the structural alignment. The TCR-CD3 structure is shown in the same orientation as that in (A). (C) Structure comparison between TCR-CD3 and pMHC-bound TCR αβ. The extracellular domains of TCR αβ from the TCR-CD3 complex were used as the template for the structural alignment. The antigen peptide is shown red

Several models hypothesize that pMHC or antibody binding to TCR α/β allosterically induces conformational changes in the CD3 subunits, thus exposing their intracellular signaling tails for phosphorylation by the Lck kinase and initiating signaling (Schamel et al., 2019). Unexpectedly, however, structural comparison indicated that pMHC binding induces no substantial conformational changes in the TCR-CD3 complex (Fig. 1B). Similar results were also obtained from structural studies of the ECDs of TCR α/β (Baker et al., 2000; Yin et al. 2012). Furthermore, the OKT3 antibody and pMHC are differently positioned for interaction with the TCR-CD3 complex (Fig. 1B and 1C), though they activate the same TCR pathways. As noted by the authors, the possibility still remains that ligand-induced oligomerization or clustering for TCR triggering. The ice cream-like structure, however, seems incompatible with TM domain-mediated oligomerization of the TCR-CD3 complex, although TM-mediated dimerization of two tilted (with respect to the PM) TCR-CD3 molecules is possible. It might be that oligomerization mediated by the ECDs further triggers conformational changes that are transmitted into the intracellular signaling domains of CD3. But TCR-CD3 oligomerization appears dispensable for TCR triggering, because monomeric agonist pMHCs anchored to a surface are sufficient to induce TCR activation (Ma et al., 2008).

Numerous studies support conformational changes in the ECDs, TMs and the intracellular tails of TCR-CD3 during activation (Schamel et al., 2019). Then how does the cryo-EM structure fit with these data? As noted by the authors, the pMHC-bound TCR α/β used for the alignment only contains the ECDs. Thus, it cannot be excluded that conformational changes occur to the ECDs of the full-length TCRα/β upon ligand binding. Additionally, lipid compositions have a role in governing the conformational states of TCR-CD3, as cholesterol binding to the TCRβ TM region was shown to lock the complex in an autoinhibited conformation (Swamy et al., 2016). Therefore, it remains to be determined whether the cryo-EM structure determined in the digitonin detergent represents a de facto resting state. The assembly of the TM segment of the TCR-CD3 complex can be compared to piston (formed by the two TM helices of TCRα/β) in cylinder (formed by the six TM helices of CD3). This seemingly agrees with the mechanical force-based models on TCR triggering (Schamel et al., 2019; Ma et al., 2007). However, piston-like movement of the two TM helices as proposed in the mechanosensor models would result in disruption of the functionally important ionic interactions formed within the TM segment of the complex (Call et al., 2002). A possible scenario might be that ligand induces transient reorientation of the TCR α/β relative to its associated CD3 subunits in their TM helices, thus enabling changes in the intracellular tails of CD3 for phosphorylation. This would agree with the notion that the TCR-CD3 complex cycles between different conformations during action (Schamel et al., 2019). Capturing of such changes, however, may not be easily amenable to structural approaches due to their transient nature. It is also possible that ligand binding may alter the orientation of the whole TCR-CD3 complex with respect to the PM as previously suggested (Kuhns et al., 2006). Ligand-induced segregation and/or redistribution of TCR-CD3 were proposed for TCR triggering (van der Merwe et al., 2000; Horejsi, 2005). How the cryo-EM structure of TCR-CD3 fits with these models remains unclear. Nonetheless, the structure provides a template to validate or disprove the multiple TCR triggering models.

The elucidation of the complete human TCR-CD3 complex structure at an atomic resolution represents a milestone in our understanding of TCR biology. The structure not only revealed the assembly mechanism of the TCR-CD3 complex but also provided information for therapeutic engineering of T cells. The successful reconstitution of a complete TCR-CD3 complex *in vitro* opens up new avenues for further dissection of the mechanisms underlying TCR signaling. It is expected that in the future similar strategies can be employed to reconstitute TCR complexes containing their interacting partners or complexes under different conditions. Biochemical and structural characterization of these protein complexes will undoubtedly reveal more exciting information on our understanding of the molecular basis of T cell-mediated immune responses and more rational design of the therapeutically important TCR-CD3 complex.

## Notes

The work supported by the Alexander von Humboldt Foundation (Humboldt Professorship of Jijie Chai). Jijie Chai declares that he has no conflict of interest.
